# Clinical and Radiological Features of Patients With Pulmonary Cryptococcosis in a Hospital of North China

**DOI:** 10.7759/cureus.8061

**Published:** 2020-05-11

**Authors:** Liling Liang, Ping Cong, Yueming Wang, Zhixin Liang

**Affiliations:** 1 Department of Respiratory Medicine, First Medical Centre, Chinese People's Liberation Army General Hospital, Beijing, CHN; 2 Department of Medical Records Management, the First Medical Centre of Chinese People's Liberation Army General Hospital, Beijing, CHN; 3 Department of Respiratory Medicine, the First Medical Centre of Chinese People's Liberation Army General Hospital, Beijing, CHN

**Keywords:** pulmonary cryptococcosis, hiv-negative, immune status, diagnosis, treatment

## Abstract

Objective: The increasing incidence has led to more focus on pulmonary cryptococcosis in HIV-negative patients. We conducted a retrospective analysis of pulmonary cryptococcosis to understand the clinical characteristics, imaging features, diagnosis, treatment and prognosis of this disease in HIV-negative patients in respiratory department of a tertiary hospital in north China.

Method: We identified retrospectively those diagnosed with pulmonary cryptococcosis in the first medical center of Chinese People’s Liberation Army General Hospital since 2009 to 2019. The clinical and image data were collected and analyzed.

Results: The study involved 34 patients with pulmonary cryptococcosis. All patients were diagnosed by histopathology. It accounted for 0.93‰ of the total number of patients admitted to respiratory department in the same period. The mean age was 49 years; 26 patients (76.5%) were male. Patients were predominantly immunocompetent (30, 88.2%), and there was no underlying disease for most patients (26, 76.5%). The most frequent symptoms were cough and expectoration. Fourteen (41.2%) patients showed no symptom or sign. Multiple lesions (21, 61.8%) and subpleural lesions (23, 67.6%) were the most common. Nodule was the most common abnormality on chest computed tomography image. Eight (23.5%) patients received serum Cryptococcus capsular polysaccharide antigen test, and seven patients showed positive result. All patients recovered after antifungal treatment.

Conclusions: Most patients of HIV-negative pulmonary cryptococcosis were mainly immunocompetent patients younger than 60 years without underlying diseases. There was lack of specificity in clinical manifestations and imaging findings. The prognosis of pulmonary cryptococcosis in HIV-negative patients was good.

## Introduction

Cryptococcusis is a kind of encapsulated yeast found widely in the environment especially in soil, pigeon dropping and rotting wood, with more than 70 identified species of which Cryptococcus neoformans and Cryptococcus gattii are commonly known to cause diseases in humans [1]. Cryptococcosis is an infectious disease with varied clinical presentations, while clinical disease is preferentially established in lung and central nervous system [2]. Pulmonary cryptococcosis, first described in 1955, is characterized by pulmonary infiltration and granuloma, of which the incidence has been increasing year by year with the enlargement of immunocompromised people, including patients with HIV infection, malignant tumors, organ or stem cell transplantation and immune connective tissue diseases [3]. As reported in a 10-year multicenter retrospective study in China, pulmonary cryptococcosis ranked third in the pulmonary mycosis (16%) [4]. In addition, it is noteworthy that patients suffering from pulmonary cryptococcosis reported in China were more commonly HIV-negative and immunocompetent in recent years [5,6]. Nevertheless, there is limited data on HIV-negative patients with pulmonary cryptococcosis in north China.

We conducted a retrospective clinical study of HIV-negative patients with pulmonary cryptococcosis in respiratory department in a tertiary hospital in north China, aiming to improve the knowledge of the demographics, clinical characteristics, radiological features and treatment of these patients.

## Materials and methods

Study design and patient recruitment

This study recruited HIV-negative patients with pulmonary cryptococcosis diagnosed by histopathology in the respiratory department of the first medical center of Chinese People’s Liberation Army General Hospital (PLAGH) in Beijing, China from December 1, 2009 to December 1, 2019. The electronic medical records were systematically reviewed for information regarding gender, age, underlying diseases, status of immunosuppression, history of environmental exposure, clinical symptoms and signs, laboratory findings, radiological characteristics, diagnosis, treatment and outcome. 

Definition

Patients were identified immunocompromised if they had any of the followings: (1) receipt of corticosteroid therapy in the past three weeks; (2) receipt of immunosuppressive agents at disease onset or in the past three months; (3) receipt of hematopoietic stem cell, bone marrow or solid organ transplantation; (4) presence of neutropenia at disease onset or in the past two months; (5) decreased natural killer cell counts in childhood; (6) inherited severe immunodeficiency. 

Statistical analysis

Statistical analyses were performed using IBM SPSS Statistics for Windows, Version 22.0. (Armonk, NY: IBM Corp.). Descriptive statistics were used to define the demographic and clinical characteristics of the patients. A chi-square test was used to determine differences between two proportions. A two-sided p < 0.05 was considered statistically significant.

## Results

Demographics and diagnosis

From December 1, 2009 to December 1, 2019, 34 inpatients with pulmonary cryptococcosis were identified. They were all HIV-negative. All patients (100%) were diagnosed by pathological examination, among which 31 (91.2%) received CT-guided percutaneous aspiration lung biopsy and three (8.8%) experienced video-assisted thoracoscopic lobectomy. It accounted for 0.93‰ (34/36,562) of the total number of patients admitted to respiratory department in the same period. The mean age of the patients was 49 years (range, 19-77 years), with the majority (30, 88.2%) of the patients younger than 60 years. Twenty-six patients (76.5%) were male, with the male-to-female ratio being 3.25:1. Of all the 34 patients, immunocompetent patients (n=30) accounted for 88.2% while four patients (11.8%) were considered immunocompromised with at least one predisposing condition. Eight patients (23.5%) were documented with underlying diseases, of which the most common was diabetes mellitus (4, 11.8%), followed by rheumatoid arthritis (2, 5.9%). There was no history of environmental exposure for most patients (97.1%), and only one (2.9%) patient had the history of staying in moist environment and close contact with soil. 

Clinical manifestations

As shown in Table 1, the most frequent symptoms were cough (11, 32.4%), expectoration (5, 14.7%), fever (3, 8.8%) and chest pain (2, 5.9%). Fourteen (41.2%) patients were asymptomatic and detected by radiological examinations incidentally. There was no statistically significant difference between patients with and without underlying diseases regarding all the clinical manifestations documented (p<0.001). There was no patient presented with central nervous system symptoms such as headache, nausea/vomiting and seizure.

**Table 1:** Demographics and clinical characteristics of patients with pulmonary cryptococcosis. SD, standard deviation.

Characteristics	Patients With Pulmonary Cryptococcosis (N=34) (n, %)
Patient demographics	
Age (years), mean ± SD (range)	49 ± 14 (19-77)
Male sex	26 (76.5)
Underlying diseases	
None	36 (76.5)
Diabetes mellitus	4 (16.1)
Rheumatoid arthritis	2 (5.9)
Lung transplantation	1 (2.9)
Primary nephrotic syndrome	1 (2.9)
Symptoms	
None	14 (41.2)
Cough	11 (32.4)
Expectoration	5 (14.7)
Fever	3 (8.8)
Chest pain	2 (5.9)
Dyspnea	1 (2.9)
Central nervous system symptoms	0 (0)
Probable environmental exposure history	1 (2.9)

Radiological characteristics and serum cryptococcal antigen test

The chest CT findings of 34 patients were analyzed (Table 2).

**Table 2:** CT image characteristics of patients with pulmonary cryptococcosis.

Characteristics	Patients With Pulmonary Cryptococcosis (N=34) (n, %)
Lesion location	
Right lung	14 (41.2)
Left lung	8 (23.5)
Bilateral lung	12 (35.3)
Subpleural areas	23 (67.6)
Lesion distribution	
Solitary	11 (32.3)
Multiple	21 (61.8)
Diffuse	2 (5.9)
Lesion patterns	
Nodule	
1-5 cm	14 (41.2)
0.3-1 cm	9 (26.5)
Mass (>5 cm)	1 (2.9)
Lobar or segmental consolidation	7 (20.6)
Patchy shadow	3 (8.8)
Cavitation	1 (2.9)
Halo sign	1 (2.9)
Air bronchgram	3 (8.8)
Spiculation	1 (2.9)

**Figure 1:**
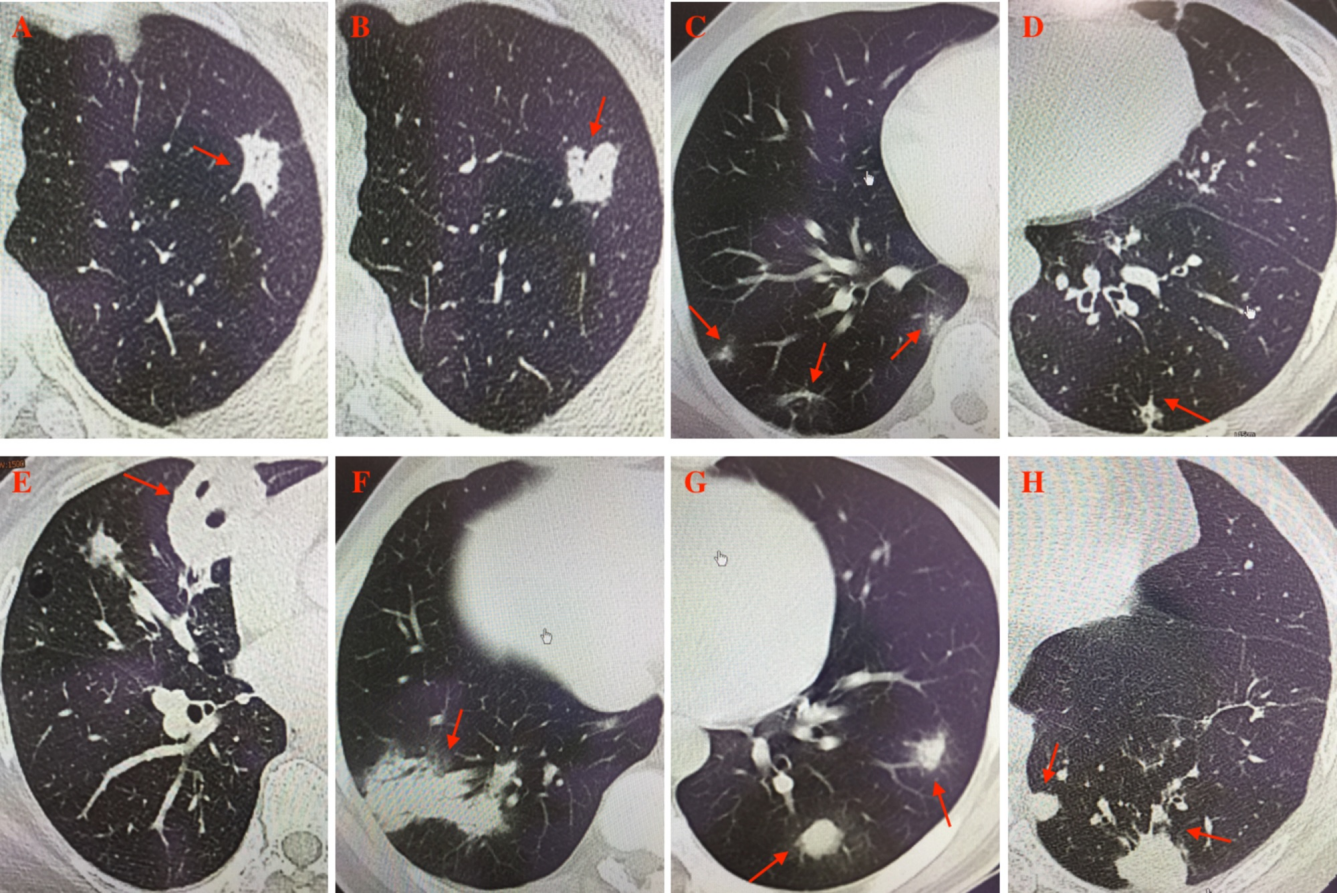
Chest CT of the patients. A: nodule with spicule sign in the left upper lobe. B: nodule with lobulated sign in the left upper lobe. C: multiple patchy shadow. D: small nodule with speculation and pleura retraction sign. E: mass with cavitation in the right middle lobe. F: consolidation with air bronchogram in the right lower lobe. G: multiple nodules with halo sign in the left lower lobe. H: multiple nodules with irregular reticular appearances and satellite lesions in the left lower lobe.

Lesions of the patients were most commonly located in the right lung (14, 41.2%), followed by bilateral lung (12, 35.3%) and left lung (8, 23.5%). Lesions were mostly located in the subpleural areas (23, 67.6%). Multiple lesions accounted for 61.8% of the cases, while solitary lesion and diffuse lesions occurred in 32.3% and 5.9% of the patients, respectively.The most common lesion pattern was nodules (23, 67.6%), particularly those with the diameter of 1-5 cm (14, 41.2%), followed by lobar or segmental consolidation (7, 20.1%) (Figure 1).

Eight (23.5%) patients received serum Cryptococcus capsular polysaccharide antigen (CrAg) test and seven patients (87.5%) showed positive result. 

Histopathology

**Figure 2:**
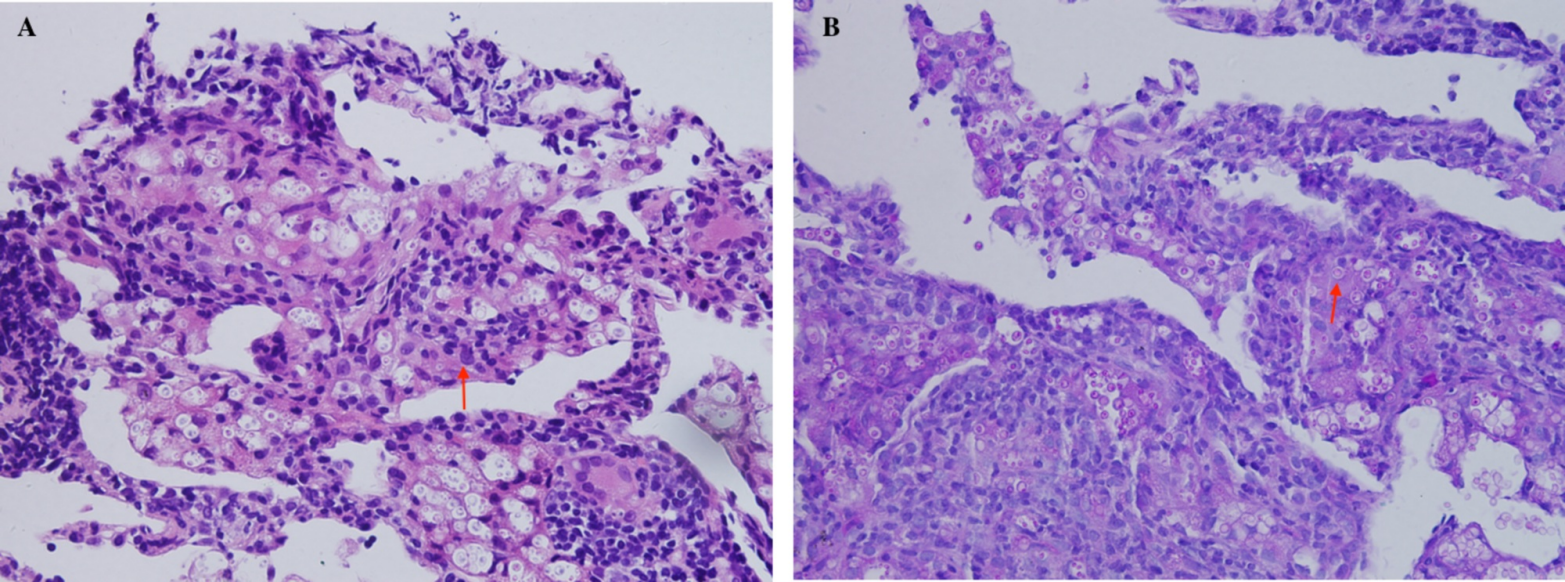
Histopathology of one case. A: hematoxylin and eosin (H&E) staining shows encapsulated yeasts (4-7 um) (×400). B: periodic acid-Schiff (PAS) staining of the yeast cells (×400).

All patients with pulmonary cryptococcosis were confirmed by pathological examination. Histological staining with hematoxylin and eosin (H&E) and periodic acid-Schiff (PAS) demonstrated narrow-based budding yeasts, usually surrounded by thick capsules in the lung tissue. Figure 2 showed the histopathology of one case.

Treatment and outcome

All patients (100%) received antifungal treatment against pulmonary cryptococcosis. Three patients experienced video-assisted thoracoscopic lobectomy and received treatment with fluconazole for three months after surgery. The other 31 patients received fluconazole, voriconazole or itraconazole after diagnosis. One patient received combination therapy with fluconazole and amphotericin B. The median treatment duration was nine months (range, 6-12 months). All patients recovered after antifungal therapy, and no patient suffered severe adverse of drugs except five cases of mild liver dysfunction and one case of subtle ocular side effect of voriconazole.

## Discussion

We presented epidemiological data on pulmonary cryptococcosis in HIV-negative patients in the respiratory departmentof a tertiary hospital in north China. Patients recruited in our study accounted for 0.93‰ of the total inpatients in our department during the same period, which was significantly lower than previously published reportsin China [7,8]. The incidence in south China and east China, which located in the tropical or subtropical regions with warm and humid climate, was much higher than that in north China. This indicated that the geographical distribution of Cryptococcus was probably related to latitude and climate. 

Similar to previous reports, the ratio of male-to-female patients was 3.25:1 in our study, which was considered possibly relevant to the differences in the immune system and physiology between male and female [9]. Pulmonary cryptococcosis can occur at any age. The patients in this study ranged in age from 19 to 77 years with the mean age of 49 years, among whom 88.2% were younger than 60 years, suggesting that the majority of HIV-negative patients with pulmonary cryptococcosis in China were young or middle-aged. 

Cryptococcosis mainly occurs in patients with immunodeficiency such as acquired immune deficiency syndrome, organ transplantation and autoimmune diseases, while immunocompetent patients accounted for only 17%-35% of patients with cryptococcosis in the studies of the United States and France [10,11]. On the contrary, patients in this survey were predominantly immunocompetent (88.2%) in accordance with the previous studies in China, which may indicate that Chinese people are more susceptible to cryptococcal infection [12]. Genetic factors probably contribute to such difference, which remains to be further investigated [13,14]. Underlying diseases involved 23.5% of the patients in this study, of which the most common was diabetes mellitus and rheumatoid arthritis.

The clinical manifestations of pulmonary cryptococcosis can range from asymptomatic infection to life-threatening pneumonia that can progress to acute respiratory distress syndrome [15]. The most frequent symptoms of the patients were cough, expectoration, fever and chest pain, while 41.2% patients were asymptomatic. Lack of specificity in the clinical manifestations may lead to delayed diagnosis or misdiagnosis. 

Since many cases were asymptomatic and detected by radiological examinations incidentally, it was necessary to investigate the radiological features of pulmonary cryptococcosis. Chest CT was commonly used in the diagnosis for pulmonary cryptococcosis of which the manifestations could be diverse. The most common lesion pattern was pulmonary nodules (67.6%), which should be distinguished from tumor and other diseases with space-occupying lesions. We found that pulmonary lesions were predominantly unilaterally and peripherally distributed. CT or ultrasound-guided percutaneous aspiration lung biopsy may be a powerful diagnostic method for pulmonary cryptococcosis. All patients except three cases diagnosed by thoracoscopic lobectomy were diagnosed by CT-guided percutaneous aspiration lung biopsy in current study. 

It was widely recognized that CrAg, a distinctive pathogenic substance of cryptococcosis, played an important role in the diagnosis of cryptococcal infection as well as the monitoring of disease activity [16]. Detection methods widely used in clinic include colloidal gold immunochromatography assay, latex agglutination test and enzyme-linked immunosorbent assay. In this study, eight patients took serum CrAg test and seven patients showed positive result. It was regret that most patients did not receive the test because it was not carried out in our center for many years.

To date, there is no prospective data available on the treatment of pulmonary cryptococcosis in China. Fluconazole was widely used in the treatment of pulmonary cryptococcosis based on its excellent activity against Cryptococcus and low toxicity [17]. As expected, it showed high efficiency in this study. All patients showed successful clinical outcome with achieving complete or partial response. 

## Conclusions

This study was the first clinical investigation on pulmonary cryptococcosis in north China. It presented the incidence, clinical and imaging characteristics, treatment and prognosis of pulmonary cryptococcosis in a large general hospital in north China. However, there were many limitations in this study. First, the data were from a single center, which cannot reflect the overall situation of pulmonary cryptococcosis in northern China. Second, the cases were all diagnosed by pathology and lack of evidence of microbial culture. The significance of this study for clinical guidance from the perspective of microorganism was limited. We hope that more large-scale and perfect clinical researches can provide more meaningful results in the future.
